# Historical applications of induced sterilisation in field populations of mosquitoes

**DOI:** 10.1186/1475-2875-8-S2-S2

**Published:** 2009-11-16

**Authors:** David A Dame, Christopher F Curtis, Mark Q Benedict, Alan S Robinson, Bart GJ Knols

**Affiliations:** 1 Entomological Services, 4729 NW 18th Pl, Gainesville, FL 32605-3425, USA; 2 London School of Hygiene and Tropical Medicine, UK; 3 Entomology Unit, FAO/IAEA Agriculture and Biotechnology Laboratory, IAEA Laboratories, A-2444 Seibersdorf, Austria; 4 Div. Infectious Diseases, Tropical Medicine & AIDS, Academic Medical Center, F4-217, Meibergdreef 9, 1105 AZ Amsterdam, The Netherlands and K&S Consulting, Kalkestraat 20, 6669 CP Dodewaard, The Netherlands

## Abstract

Research on sterile mosquito technology from 1955 to the 1980s provided a substantial body of knowledge on propagation and release of sterile mosquitoes. Radiation sterilisation and chemosterilisation have been used effectively to induce dominant lethality and thereby sterilise important mosquito vectors in the laboratory. Experimental releases of chemosterilised males provided complete control of *Anopheles albimanus *in a small breeding population (14-15 sq km) in El Salvador. Releases of radiation sterilised males failed to control either *Aedes aegypti *or *Anopheles quadrimaculatus *in the USA. Releases of radiation-sterilised and chemosterilised male *Culex quinquefasciatus *in the USA and India were successful in some instances. Development of genetic sexing systems for *Anopheles *and improved physical separation methods for *Culex *have made it possible to rear and release males almost exclusively (> 99%) minimizing the release of potential vectors, the females. Factors that affected efficacy in some field programmes included reduction of competitiveness by radiation, immigration of fertilized females from outside the release zones, and inability of laboratory-bred males to perform in the wild. Despite significant progress, institutional commitments to carry the process further were generally lacking in the late 1970s and until recently. Now, with renewed interest and support for further assessment of this technology, this paper summarizes the current knowledge base, prioritizes some areas of investigation, and challenges scientists and administrators to maintain an awareness of progress, remain realistic about the interpretation of new findings, and make decisions about the sterile insect technique on the basis of informed scientific documentation. Areas recommended for priority research status include the establishment of genetic sexing mechanisms that can be transferred to other mosquito species, re-examination of radiation sterilisation, aerial release technology and mass rearing.

## Background

The first successful use of the sterile insect technique (SIT), in the early 1950s, involved the New World screwworm *Cochliomyia hominivorax*, a serious veterinary pest of the western hemisphere. This demonstration of a very challenging new method of pest control led almost immediately to attempts to develop similar approaches to control public health pests, especially mosquitoes. Forty years later, when the New World screwworm had been eliminated from all of North America, Central America and Panama, research on mosquito SIT had dwindled from a major international thrust to a limited academic arena. This paper reviews the main research endeavours that took place from the 1950s to the 1980s, and describes the resulting knowledge and experience that now provides the informational baseline for this renewed interest in mosquito SIT.

## Major field trials with released sterile mosquitoes

### Theory and application

Sterile insect technique (SIT) has been operational since the late 1950s. Despite the many SIT successes with several insect species during the last four decades, there are still scientists and administrators who do not fully understand the principles or the limitations of the technology. Yet the practitioners of the technology report that as a component of area-wide integrated pest management (AW-IPM) programmes, SIT is currently saving billions of dollars annually in commerce, export markets, reduced environmental impact from pesticides and reduced losses to pests. The initial beneficiaries of this technology were the cattle industry and the wildlife that were spared the ravages of the New World screwworm, now eliminated from North and Central America south through Panama.

Knipling's theories [1] focus on AW-IPM concepts, under which SIT can target both large and small areas of pest infestation for elimination, suppression or prevention. Depending upon the specific objective and the population characteristics of the target species, SIT can very rarely stand alone and is more likely to be used in combination with prior suppression and/or complementary concurrent control activities. Initiation of AW-IPM programmes, in the USA for example, usually entails governmental and user (shareholder) agreements, which may require formal referenda in which two-thirds of the shareholders must agree. Prior planning for such programmes includes in-depth economic analysis based on capital investment, long-term costs and benefits, and comparison to conventional approaches. Public education is a key component of AW-IPM programmes, which usually include several complementary modes of control. Perhaps the most important aspect of SIT is the realization that it is suitable for only a select group of pests and situations - determined by pest biology, geography, economics and political climate.

Some key misunderstandings have led prominent scientists to underestimate the flexibility and utility of SIT. For example, the target insect need not be monogamous. It is important, however, that sperm of sterilised males be competitive with sperm of the wild males. It is not mandatory that a high ratio of sterile males be attained at the onset, although this certainly would be a desirable situation. The necessary level of over-flooding depends on the biology of the insect, the competitiveness of the released insect and the complementary methods of control that are being considered. It is not absolutely necessary that the infestation be isolated from other sources of the pest, but when isolation is not possible, the programme must have the capability of eliminating the influence of immigrating fertilized females. It is not absolutely necessary to eliminate the target species from an experimental plot to demonstrate that the technique works. Carefully planned research can show what the specific impacts of the releases and other factors, such as immigration, have been. With this information, managers can predict with a high level of probability what can be achieved in operational programmes and avoid the usually impossible task of finding the perfect field plot for proving that the system works.

Perhaps most confusing is the relationship between numerical release requirements and the biotic (reproductive) potential of the pest. Biotic potential usually varies seasonally and may be density dependent. If the effective over-flooding sterile male ratio is 9:1, and the biotic potential (rate of increase R_0_) of the target population is five-fold per generation, then the second generation natural population will be only 50% of the initial density (Table 1). However, if the biotic potential is 0.5 (50% decline in population density per generation), the same release ratio will reduce the natural population to 5% of the initial density in the second generation. Rates of increase vary with location, season and environmental conditions. These and other quantifiable parameters are determined during the research and pilot study phases of SIT development, and then further adjusted in the operational phase. If the initial release ratios are sufficient to reduce R_0 _to less than 1.0, continued release of the same number of insects will cause population decline. In subsequent generations, the increasing level of induced sterility will eventually lead to elimination of the wild population. Computer models are available for assessing SIT strategies and outcomes [2,3]. These simulation models can provide valuable insight for programme planning and conduct, but generally provide the basic framework rather than the specific details for a given pest.

**Table 1:** Theoretical population trend with nine sterile males released for each initial fertile wild male per generation with a 5-fold rate of increase (after Knipling [1])

**Generation**	**Density of fertile males**	**No. released males/generation**	**Ratio of sterile to fertile males**	**Population density**
1	1,000,000	9,000,000	9:1	500,000
2	500,000	9,000,000	18:1	131,580
3	131,580	9,000,000	68:1	9,535
4	9,535	9,000,000	944:1	50
5	50	9,000,000	180,000:1	0

The SIT approach involves sophisticated technology and experienced staffing is required to effectively manage the rearing processes and distribution of sterile insects. Use of live organisms to control wild populations of the same species can generate unexpected problems. Knowledgeable, well-trained leadership and staff continuity are prerequisites. As an example of what can be accomplished, there are now about 20 fruit fly rearing facilities around the world, one of which currently produces over three billion sterile male *Ceratitis capitata *pupae weekly for distribution to contracted clients.

Large programmes may continue for decades. The New World screwworm programme took more than 40 years to gradually eliminate the infestation in North and Central American and establish a barrier zone in Panama. Administrators must be convinced about SIT needs and objectives and be dedicated to extended programme continuity and fiscal support. The cost-benefit analysis must justify the long-range commitment.

### Mosquito SIT programmes

For public health pests, SIT has not reached the operational level, but has been a subject of extended research since the mid-1950s. Mosquitoes that transmit pathogens to humans received much attention during the two decades following Knipling's initial SIT successes. There is an excellent review of each of these efforts which includes objective interpretations of the more ambitious studies cited below [4].

### Ionizing radiation

The first sterile mosquito releases were conducted in 1959-1960 by the United States Department of Agriculture (USDA) in South Florida with males that emerged from radiation-sterilised (120 Gy) *Anopheles quadrimaculatus *pupae [5]. Releases totalled ca 32,000 (over three months) and 300,000 (over nine months) in 1959 and 1960, respectively. Lack of sterility in the wild population led to studies that implicated a changed mating behaviour of the colonised male mosquitoes leading to reduced incidence of mating with wild females and the consequent failure [6].

Ineffectiveness of released radiation-sterilised (110-180 Gy) *Aedes aegypti *in Pensacola, Florida for two mosquito seasons (1960-1961) by the Centers for Disease Control (CDC) [7] was later attributed to reduced competitiveness caused by irradiation in the pupal stage. Releases totaled 3.9 million in 1960 (four months) and 6.7 million (six months) in 1961.

Effective releases of irradiated (60-120 Gy) *Culex quinquefasciatus *were conducted by the World Health Organization/Indian Council of Medical Research (WHO/ICMR) in India and the USDA in Florida between 1967 and 1974 [8,9]. These studies confirmed previous laboratory findings that pupal irradiation can be detrimental; but somatic damage was lower with older pupae than with 0-24 h old pupae and competitiveness was reported to be unaffected when one day old adults were irradiated. Releases of sterile males generally ranged from 9,000-15,000 daily in several separate experiments.

In 1980, the first phase of a two year seasonal release study in California, *Culex tarsalis *males were determined to be fully competitive following irradiation (60 Gy) as adults [10]. However, in the 1981 releases, there was a significant inability of the sterile males to seek out and mate with wild females. Lack of control despite adequate over flooding ratios was attributed to assortative mating brought about by selection during colonisation a few months prior to the 1981 releases. A total of 71,000 and 85,000 sterile males were released in 1980 and 1981, respectively.

### Chemosterilisation

Several alkylating agents and aziridinyl compounds have been tested as mosquito sterilants. Successful laboratory ([11], for example) and small-scale field ([12], for example) studies of these chemicals led to large scale field assessments with *Cx. quinquefasciatus *in India by the WHO/ICMR Unit in New Delhi, *Anopheles albimanus *in El Salvador by USDA and CDC at the Central America Malaria Research Station in San Salvador, and other species elsewhere [4].

As part of a broad WHO/ICMR experimental programme on genetic control options for mosquitoes, chemosterilisation studies were initiated with *Cx. quinquefasciatus *and *Ae. aegypti *in New Delhi around 1971. Thiotepa was selected from several sterilants assessed. Sterile males treated as pupae and released as pupae or adults in several separate field studies were found to be competitive and capable of inducing sterility in wild mosquito populations [13]. Up to 300,000 sterile males were released daily. However, the study areas were subject to immigration of fertilized females from other breeding sites, whose fertile egg masses impacted severely on the level of sterility and control ultimately achieved. Nevertheless, the suitability of the chemosterilised males was confirmed. The breadth of the investigations touched on many essential aspects of the methodology of conducting control operations with sterile mosquitoes.

In El Salvador, experimental releases of chemosterilised male *An. albimanus *were initiated at the beginning of the major breeding season in 1971 at Lake Apastapeque, about 40 km from San Salvador [14,15]. Pupae, sterilised by 60 min immersion in an aqueous bisazir chemosterilisation solution [16], were placed in emergence pans in wooden shelters in natural breeding areas. Starting with an estimated over-flooding ratio of only 2:1, the wild mosquito population was reduced to the point where neither immature stages nor adults could be detected after about five months, suggesting a high level of competitiveness and dispersal ability among the released insects. The rapid increase in vector density normally associated with the malaria transmission season was prevented by these releases. Up to 40,000 sterile males were released daily at the 14-15 km^2 ^site.

This experiment was followed by a larger scale (150 km^2^) pilot study to test the capability of releasing one million sterile males daily in an integrated programme for control of *An. albimanus *in a mountainous coastal region [17]. To minimize the potential for transmission by released females, special attention was given to improved separation of the sexes (separation by size alone had yielded 86% males and 14% females in the Lake Apastapeque trials). A dual sexing system was therefore adopted. Following separation by pupal size and sterilisation, the emerged adults were held in cages for 2-3 days and then offered a bloodmeal containing insecticide (malathion). After the females had fed and died, the males were collected and released. However, these males were not competitive and, furthermore, only ca 40% of the males produced actually survived long enough to be released. Field studies then revealed that sterile males that had emerged from pupae placed in the field were more competitive, whereas males held in cages for one day were less competitive, and males held in cages for 2-3 days were unable to induce sufficient sterility into the indigenous population.

To attain maximum production and competitiveness, the colony strain was replaced by a strain with a chromosome translocation linking propoxur resistance to the Y-chromosome [18]. Exposure of eggs to propoxur killed the females but not the males. Twice as many males could then be produced from each rearing unit and released in the pupal stage. Males of this genetic sexing strain (MACHO) performed very well in the field, reducing the wild population in a small (20 km^2^) experimental block to a fraction of its prior density while the population in the untreated control zone increased several fold. The releases were then integrated with larval control measures and the population was further reduced to one-tenth of its original density (97% control in four months). At this stage of the research, the special support funds had been exhausted and El Salvador was in a state of civil disruption. Studies were discontinued.

### Comment

During the two decades following the initial mosquito SIT experimentation in south Florida with *An. quadrimaculatus *there had been a great deal of progress. Means of producing highly competitive sterile male mosquitoes had been developed. Many technical barriers had been hurdled, but institutional commitments to carry this process to the next step were generally lacking.

## Factors critical to the deployment of sterile male mosquitoes

Readers should now realise that to date there have been no operational-level mosquito SIT programmes. However, the intensity of prior scientific research has produced valuable information for future activities, but it is evident that much still needs to be done to determine if and how well SIT will meet the needs of major disease management programmes. The SIT experimentation and preparation process tends to be long-term and expensive.

Nevertheless, one should not expect that research in itself will answer every question related to mosquito SIT. There is no complete guarantee of operational success regardless of the amount of research conducted. Decision makers will need to interpret the findings of the research community and determine the likelihood of achieving the desired objectives. The challenge for researchers will be to develop sufficient information for decision makers to rationally weigh the benefits of SIT against the risks and cost of implementation. However, it should be assumed that some important questions regarding each specific SIT programme will not be answered until operational level programmes are underway. In the following sections some of the more important, but perhaps less apparent, aspects of SIT implementation are addressed.

### Surveillance

Surveillance is an essential component of SIT. The ecology and biology of the target species throughout a proposed release area must be well understood. Seasonal patterns of mosquito distribution and density estimates are integral parts of the database that is used to plan strategies and initiate actions. Surveillance must continue throughout the programme and for an unspecified duration after the wild population can no longer be detected. This activity provides the data required to determine if and when releases should be terminated. A significant portion of the budget will be dedicated to surveillance for the purpose of documenting the extent and nature of the pre-release populations, monitoring progress during the programme and confirming the status in the post-release phase.

### Population dynamics

Determining population trends and establishing appropriate over-flooding ratios is not an exact science. This is because R_0 _(rate of increase, explained previously) changes seasonally, geographically, and in response to a variety of abiotic and biotic factors. Since sterile male efficacy is in part dependent on the over-flooding ratio achieved in each generation, estimates of changes in mosquito abundance are critical components of planning and execution. As an example, in the Lake Apastapeque study, R_0 _was found to range from 0.4-4.8 [19]. SIT is more efficient when R_0 _is small than when it is large because fewer released insects are required to achieve the same result. In that study, releases were initiated just before the end of the dry season when the wild mosquito R_0 _and abundance were low in order to optimize the impact of the sterile males that were available for release and to avoid the need for a prior suppression effort. The timing of this action prevented the rapid increase in vector density that was expected to result soon because of the onset of a period of large R_0 _values.

Density dependent survival has been observed in experimental mosquito SIT projects. This phenomenon occurs when population density drops sufficiently to alleviate conditions that normally restrict population growth. For example, when *Culex *larval habitats become overcrowded and an external stress factor (including SIT-induced sterility) causes a rapid decline in density, the R_0 _may respond by exceeding - even doubling - its normal maximum. An increase in R_0 _to 10 (normal maximum = 5x) was observed in an experiment with *Cx. quinquefasciatus *in Florida [20], but this density dependent phenomenon was not observed in the *An. albimanus *population at Lake Apastapeque [19]. However, when it does happen, increased numbers of sterile male may be required.

When population levels are low, SIT is most efficient because it is easier to achieve the over-flooding ratios necessary to initiate population decline. Thus, it is advantageous to use alternative control options to reduce mosquito density to levels that are compatible with the numerical release potential, and it is most beneficial to do so when the natural R_0 _of the target population is at its lowest. Fortunately, current mosquito management practices are compatible with SIT, especially those that do not specifically target the released males.

### Competitiveness

When released insects are not fully competitive, the release numbers must increase to compensate for the deficiency. For example, when competitiveness is 0.5, twice as many sterile males are needed as when competitiveness is 1.0. Competitiveness estimates are usually derived in laboratory or field cages by introducing known numbers of sterile and fertile males to compete for virgin females. A standard calculation method is used to determine competitiveness [21]. Competitiveness values range from zero to one, and they are a measure of the ability of the sterile males, in competition with wild males, to successfully mate with wild females. Experiments to measure competitiveness can be conducted in natural mosquito habitats when wild mosquito density is low by releasing known numbers of each male type along with marked virgin colony or wild females. Fertility of recaptured marked females indicates the level of competitiveness. In the case of the MACHO strain, the observed competitiveness in this type of field study was high - 0.78-0.80 [22].

In practice, effectiveness of the released male involves much more than competitiveness. Population reduction is a function of the average effective released male:wild male ratio. This is influenced by, e.g., frequency and distribution of releases, effect of release techniques on competitiveness, longevity of the released males and their ability to disperse and locate mates, distribution of the wild population and losses to predation. These parameters are not easily measured. Managers need to custom-fit the release to match known habitat preferences, adjust for heterogeneous distribution of the wild population and other factors or simply release more sterile insects than are calculated to meet a specified ratio. As an example of the discrepancy between estimates of competitiveness and release requirements, Patterson [9] reported a competitiveness value of 0.75 in a cage study for *Cx. quinquefasciatus *males sterilised as adults, but after release the competitiveness value was estimated as 0.25-0.33. The 0.25-0.33 estimate from the field study is an indication of the released male's average effectiveness, indicating that it actually took 3-4 released males (rather than 1.3) to compete with one wild male. To achieve an effective 10:1 ratio under this condition would require a release rate of at least 30:1.

### Rearing and handling

Proven rearing methodologies exist for the daily production of millions of some mosquito species, e.g., *Cx. quinquefasciatus, Ae. aegypti *and *An. albimanus *[23-25]. Yet, there is a continuing need to develop methods of rapid colonisation that 1) minimize the adverse effects of selection [26], 2) minimize space and personnel requirements, 3) maximize the yield of hardy insects, and 4) minimize the release of female mosquitoes. Mosquito production for the WHO/ICMR and Lake Apastapeque experimental releases focused on the need to exclude females. In the WHO/ICMR project, separation of *Cx. quinquefasciatus *pupae by size yielded releases that were > 99% males [13] at the expense of considerable wastage of the males produced. In the Lake Apastapeque programme, 14% of the released insects were females, but no estimates are available for male losses during size separation. However, male production per tray was doubled, and male losses were minimized (ca 10%) after adopting the MACHO genetic sexing strain.

Although the MACHO strain made it possible to increase male releases by 3-4 fold and release extremely low numbers of females, management of the rearing process became more complex [24]. Egg viability of the strain was only 50% because of the translocation so the number of cages holding females for egg production was doubled. A separate colony had to be maintained to ensure the purity of the brood stock used for release production because of genetic recombination (0.1-0.2% per generation). Without informed management, this loss of linkage of insecticide resistance with the Y chromosome would gradually result in an increase in recombinant resistant females and susceptible males. It was necessary to purge the recombinants and replace the stock at regular intervals despite the stabilizing inversion that had been incorporated into the strain [27].

### Sterilisation methodology

Excessive levels of sterility in released males could reduce their effectiveness as mosquitoes suffer somatic damage as a result of exposure to radiation. Achieving 100% sterility might be counter-productive if it results in a substantial loss of male competitiveness (see [28] for a detailed discussion). The competitive sterile males released at Lake Apastapeque were 99.8% sterile after exposure to bisazir (females, 96.6%) [16], which was adequate to achieve control. Sterility requirements are generally related to the biotic potential of the target species. If there are data on this potential and on mating competitiveness in the field, the optimum male sterility level could be calculated. Sterility levels in females need to be considered, but in general, radiation levels that sterilise male mosquitoes also sterilise the females. Chemosterilant levels that completely sterilise males produce high levels of sterility in females. This sterility usually is permanent, lasting for the lifetime of the insect.

Radiation sterilisation and chemosterilisation offer straightforward methods to produce sterile male mosquitoes. However, ionizing radiation is known to cause somatic damage and severely reduce competitiveness when pupae are exposed to sterilising doses, but older pupae are not as sensitive. Although mosquito males irradiated as young adults have often displayed lowered competitiveness in past field trials, recent laboratory findings suggest that somatic damage may be avoidable when newly emerged adults are irradiated [28]. Chemosterilisation appears to provide the option of sterilisation without somatic damage. Concerns about the environmental fate of chemosterilant residues highlighted by laboratory bioassay of non-target predators [29] may have been answered by findings of extremely low initial residues, virtually complete degradation within 24 h post-treatment and simple bulk detoxification methods [30]. Using modern equipment and personal protection, workers involved with the sterilisation process can be protected from the hazards of radiation and chemosterilants.

### Packaging, transport, release mechanisms and strategies

Sterile mosquito releases conducted to date have relied on ground release. Relatively simple packaging, transport methodology, release containers and shelters have been devised for pupal and adult releases [31], but no work has been initiated on methods of aerial distribution. Certainly, in urban programmes ground release might suffice, but the availability of satisfactory aerial release methods could provide timelier and more effective distribution with reduced opportunity for pre-release damage to the sterile males. Production and release of millions per day will demand expedited delivery mechanisms to prevent losses in quality and competitiveness.

Scheduling of releases can be very complex, even with the availability of computerized models that incorporate geographical information systems. Continuous adult emergence is characteristic of some mosquito species most likely to be considered for SIT but little work has been conducted on determining appropriate release intervals. An important factor for scheduling release intervals is the likelihood that the average lifespan of sterile males may be shorter than wild males as has been documented with irradiated mosquitoes [8,9]. This aspect may influence release distribution patterns as much as wild mosquito density, aggregation, and released male dispersal parameters. Distribution sequence will also be dictated by geographic characteristics, with release patterns for urban settings perhaps differing from those of rural locations.

**Figure 1:**
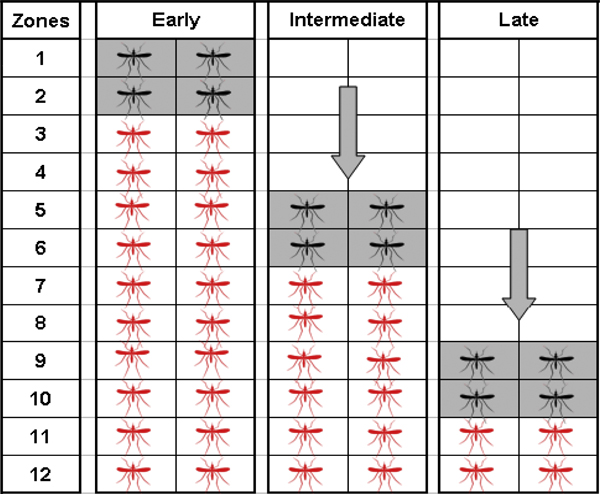
**The concept of releasing sterile mosquitoes on a "rolling front" or "rolling carpet"**. Three phases of a release programme for the same areas are shown. Red mosquitoes represent natural populations, black ones sterile release areas and open boxes disinfested zones. The spatial direction of the releases and the accompanying disinfested areas expansion are indicated by the arrows. This also illustrates the "area-wide control" concept in that there is an increasing disinfested area in return for a continuous level of effort.

SIT programmes could be faced with the options of area-wide or selective suppression of the wild mosquito population prior to initiating releases. It is conceivable that major sectors of the target area may not breed mosquitoes and that infestation foci are well defined, which could lead to selective release coverage based on the extent of the individual infestations with generous buffer zones to ensure disruption of the potential impact by immigration of fertilized mosquitoes. Alternatively, the programme could advance along a common front as population control is achieved in the area designated for the initial releases (Figure 1).

### Quality control

During the implementation of SIT programmes, there are procedures, equipment and facilities that must operate according to pre-determined standards. These standards are maintained by those responsible for the specific activities, but there also needs to be an oversight component to ensure that the standards are understood and faithfully observed. This task falls to quality control (QC). Examples of standards to monitor are: mean larval, pupal and adult weights, pupae produced/standard rearing container, sex ratio, adult longevity and sexual aggressiveness of pre and post release males, eggs per colony female, blood and food quality. To ensure that QC acts independently and is free from the influence of those groups that it oversees, QC is routinely placed between Management and Operations in the organizational hierarchy. Leadership of the QC team should have management-level authority in order to ensure objectivity and preserve the ability to act independently. Based on its findings, QC advises Operations and reports directly to the Director.

Minimal attention has been given to the possibility of mosquito escape from most SIT research-level rearing facilities, whereas in existing operational programmes located in disinfested zones, e.g., screwworm and fruit fly, facility quarantine is a major consideration. Unintentional mosquito release from either research or operational activities not only could jeopardize the success of the programme, but it could also cause significant public relations and public health problems. Fail-safe procedures are necessary components of rearing facility management to prevent unintentional escape and to ensure that intentionally released insects are sterile.

### Research

Research does not end when operational activities begin as there is a continued demand for problem solving and advance preparation. Major SIT programmes require an independent on-site research component acting in support of the programme. Problem-solving and development of supporting technology should not be the responsibility of production or release staff, although they should be active contributors to the research programme.

### Key issues

In light of the progress that has already been documented, perhaps the more pressing issues that need to be addressed in depth are:

• Stable genetic sexing mechanisms, possibly transferable to multiple species

• Optimization of radiation sterilisation, especially in the pupal stage

• Development of aerial release capability

• Optimization of rearing technology for species under consideration

• Realistic decision making by administrators with awareness of research limitations

## Current priorities

### Initial perspectives

The preceding discussion dealt with intensive studies that have provided in-depth experience with mosquito SIT. Although the feasibility of using SIT for controlling mosquitoes of public health importance has been established, the optimum strategies, logistics and economics have yet to be determined. The research phase must continue to determine if practical application of the technology is possible. Equally important is the close relation to parallel genetic and molecular approaches that could benefit from advances made in SIT, especially for propagation and distribution of live organisms. Within the framework of this paper, i.e., field studies, there is no lack of worthy objectives. The final section focuses on the primary issues that deter the implementation of mosquito SIT technology.

### Genetic sexing capability

Stable genetic sexing mechanisms need to be developed in order to prevent or minimize the release of female mosquitoes that could transmit disease. None of the physical separation procedures used to date is capable of complete separation, and all are accompanied with some level of male loss. Furthermore, the ability to mass rear only males in the production facility virtually doubles the quantity that can be sterilised and released. With greater numbers of sterile males, managers can attain more rapid control or enlarge the area of release - or both.

It may not be feasible to achieve 100% elimination of females in the mass production process, but certainly > 99% is achievable and had already been documented with the *An. albimanus *MACHO strain used in El Salvador and *Cx. quinquefasciatus *in India. If 100% is not possible, then it will be incumbent upon programme managers to justify the numbers of females that would be released based on the probability of biting nuisance and increased transmission. However, in almost all scenarios the projected number of released females would be a small fraction (perhaps less than 1-2%) of the numbers of wild females that would transmit disease in the absence of a successful release control programme. In the Lake Apastapeque programme, the releases held the wild female population at unprecedented low levels throughout the main transmission season. In future programmes, there is a strong likelihood that suppression measures used prior to the initiation of releases will reduce transmission to levels far below the potential for released females to cause an increase in normal levels of transmission.

A variety of genetic possibilities exists for creating mechanisms that would provide complete elimination of females [32]. Whichever ones reach the operational level, they hopefully will be transferable to other species so that female release would no longer be considered a limiting factor for SIT programmes.

### Radiation sterilisation

There are many reasons for using radiation to sterilise insects, but the results with mosquitoes have been mixed. Dose, stage and age have surfaced as primary parameters related to the somatic damage caused by irradiation. Ideally, pupal irradiation provides the maximum flexibility for operational releases by giving managers the option of using either pupal or adult releases - or both - depending on local circumstances. Handling can be substantially reduced by irradiating in the pupal stage, thereby reducing non-radiation causes of reduced competitiveness. It is imperative that research on radiation sterilisation address the basic causes of radiation-related somatic damage. Parameters that should receive attention include stage, age, dose rate, atmosphere (nitrogen, carbon dioxide, etc. as protectants) and temperature [28].

### Aerial distribution

Development of aerial release capability is an extremely important objective for mosquito SIT. Ground release of sterile males is not only time-consuming and relatively inefficient; it also provides opportunities for individual handling errors and creates extra stresses on the confined insects. While there is a place and a need for ground release capability, aerial applications have traditionally offered rapid, verifiable distribution and area-wide coverage. This is particularly evident in rural situations. Possible starting points might be with the research support groups for existing SIT programmes and biocontrol programmes that have experience with inundative and inoculative releases.

### Rearing technology

Major savings in SIT costs can be realized with modifications in rearing methods that reduce space, personnel and/or nutritional requirements [33]. A case in point is the MACHO strain, which doubled the rearing capacity, reduced space, personnel, and nutrition costs per million males. Small modifications such as food sources and handling procedures can also yield significant benefits. New approaches to augment traditional forms of rearing might yield results. Each species under consideration for SIT could benefit from advances made with other species, especially if the changes are transferable.

### Administrator awareness

Practitioners of SIT, whether they are in research, operations or supporting activities, need to keep in touch with upper level administrators. As indicated at the beginning of this paper, administrators need to be informed about the benefits of SIT and the long-term fiscal commitments that are required. Usually, decision makers have broad budget responsibilities, and it is essential that they be informed about as many aspects of the programme as is possible. Bring them to work sites, expose them to educational SIT videos and other media that provide different perspectives on the programme. Get them out of the office for a better first-hand appreciation of the programme benefits and strengths.

A frequently repeated problem related to initiating AW-IPM programmes is the perceived need, by both scientists and administrators, to conduct enough research to prove beyond a shadow of doubt that the approach will work. While it is of utmost importance to document a very high probability of success, the costs of mass production and experimental field releases are such that replication is not always possible and isolation of experimental areas is seldom feasible. Managers have to interpret the impact of immigration on density reduction as well as the impact of the released insects. So scientists need to be prepared to do just that - show how immigration affects the research results. By so doing, managers can make rational presentations on how well the technique worked even when complete target mosquito population elimination is not achieved and why operational SIT should be the next step - if that is the case. Another factor to consider is that to date there have been no large operational mosquito SIT programmes, and the likelihood that an operational programme will become more efficient over time and solve many known problems is predictable.

One often used and acceptable way of reducing costs without sacrificing experimental information is to use population trend data acquired in the year(s) prior to pilot releases as the untreated control data for comparison with the data acquired during the experimental period. Ideally, one would obtain both pre-intervention data and have a contemporary control, but in practice, dispensing with one of these may be unavoidable for economic reasons and experience shows that one can often do so and still obtain convincing results.

If managers are careful to collect the appropriate information (density, distribution, induced sterility levels, etc.) in both periods, it is possible to document programme impact without the need for concurrent untreated plots. The cost of absolute proof in terms of time, resources and available test plots is likely to be unaffordable. The level of efficacy can be measured by appropriate data collection and achieving results that demonstrate the next step to be reasonable.

## Competing interests

The authors declare that they have no competing interests.

## Authors' contributions

DAD wrote the paper and CFC, MQB, ASR and BGJK made subsequent contributions and edited the paper.
